# Patterns and predictors of repeated violent suicide attempts: a retrospective clinical study from an acute trauma care setting

**DOI:** 10.3389/fpsyt.2026.1736615

**Published:** 2026-02-10

**Authors:** Noemi Monika Szeifert, Lajos Balint, Xenia Gonda

**Affiliations:** 1 ELTE Eötvös Lóránd University, Doctoral School of Psychology, Budapest, Hungary; 2 Department of Sports Medicine, Semmelweis University, Budapest, Hungary; 3 HCSO Institute for Quantitative Population and Economic Research, Budapest, Hungary; 4 Department of Sociology, Faculty of Humanities and Social Sciences, University of Pécs, Pécs, Hungary; 5 Department of Clinical Psychology, Semmelweis University, Budapest, Hungary; 6 Department of Psychiatry and Psychotherapy, Semmelweis University, Budapest, Hungary; 7 NAP3.0-SE Neuropsychopharmacology Research Group, Hungarian Brain Research Program, Semmelweis University, Budapest, Hungary

**Keywords:** completed suicide, integrated psychiatric and trauma care, mental disorders, suicide attempt, violent attempt

## Abstract

**Background:**

Violent suicide attempts pose a major challenge to trauma and psychiatric services due to their high lethality and complex clinical profiles. While non-violent suicidal behaviors have been extensively studied, considerably less is known about the recurrence of violent suicide attempts. This study examined demographic and clinical factors associated with repeated violent suicide attempts in Hungary.

**Methods:**

A retrospective chart review was conducted at the Dr. Manninger Jenő National Trauma Center in Budapest among patients admitted between January 2015 and December 2024 following a violent suicide attempt. Electronic health records provided sociodemographic, psychiatric, motivational, and method-related data. Subgroup and logistic regression analyses were used to identify predictors of repetition.

**Results:**

The final sample comprised 327 individuals (222 males, 105 females; mean age = 46.95 years, SD = 19.5). Repeated violent suicide attempts were observed in 18% of females and 10% of males. Overall, 31% of violent suicide attempters had a history of a prior non-violent suicide attempt followed by a transition to violent methods. Males had approximately threefold higher odds of transitioning from non-violent to violent methods. Among suicide deaths, 76% occurred in males, and 96% were fatal at the first attempt. Personality disorders (OR = 4.15, p = 0.028), substance use disorders (OR = 2.86, p = 0.005), and sedative/hypnotic medication dependence (OR = 3.72, p = 0.009) were significantly associated with repeated violent suicide attempts, particularly among males. Polytoxicomania was associated with nearly fourfold higher odds of repetition (OR = 3.97, p = 0.0004). A history of a prior violent suicide attempt was the strongest predictor of repetition (OR ≈ 660, p < 0.000001), independent of age and sex. Acute psychotic symptoms, while not inherently motivational in themselves, emerged as the most frequently reported proxy associated with violent suicide among repeat attempters, followed by relational conflict, existential crisis, and chronic illness.

**Conclusion:**

Repeated violent suicide attempts constitute a distinct and exceptionally high-risk clinical phenotype. Prior violent attempts, male sex in the context of repetition, personality disorders and substance abuse—particularly polytoxicomania—emerged as salient risk markers. These behaviors appear to arise at the intersection of prior violent conduct and acute substance-induced psychopathological states, notably psychotic symptoms with impaired reality testing and heightened impulsivity. Early identification and integrated psychiatric care, initiated during acute trauma management and maintained throughout rehabilitation, are critical to mitigating subsequent suicide risk in this vulnerable population.

## Introduction

Suicide remains a major global public health concern, accounting for an estimated 720,000 premature and largely preventable deaths annually worldwide (1). Suicide is a complex interplay of biological, psychological, and social factors. A widely adopted conceptual distinction in contemporary suicide research differentiates between violent and non-violent suicidal behavior based on the method employed. This dichotomy has gained prominence owing to robust evidence demonstrating a strong association between the method used in a suicide attempt and the subsequent risk of death by suicide, with violent methods exhibiting markedly higher lethality and case fatality rates (2–4). Beyond its relevance for risk stratification, the classification of suicide attempts according to method is of particular importance for prevention, as it directly informs targeted intervention strategies, means restriction policies, and clinical risk assessment (3). Based on the widely used criteria proposed by Åsberg, violent suicide attempts, defined as self-directed acts with intent to die that involve high-force or mechanically traumatic methods resulting in severe bodily injury—such as hanging, jumping from heights, use of firearms, or self-inflicted stabbing—are more frequently associated with higher fatality rates than non-violent suicide attempts, which typically involve self-poisoning or intoxication and are characterized by comparatively lower immediate physical force (5–9). Hungary has traditionally exhibited some of the highest suicide rates in Europe, peaking at nearly 45 per 100,000 inhabitants in the 1980s and remaining elevated in recent years, with a reported rate of 17.5 per 100,000 in 2020 (1, 7, 10, 11).

During the COVID-19 pandemic, national data showed an increase in completed suicides in Hungary from 1,550 in 2019 to 1,705 in 2020, with all recorded cases involving violent methods (12–15). This trend highlights a key epidemiological shift and emphasizes the ongoing severity associated with violent suicide methods. A substantial body of research has examined the so-called “gender paradox” in suicidal behavior: women more often engage in non-lethal, non-violent suicide attempts, whereas men are well established to use violent methods more frequently than women, both in suicide attempts and in completed suicide, and are therefore disproportionately more likely to die by suicide (6, 16–19). Consistent with this pattern, suicidal acts have been shown to be three to four times more lethal in men than in women, a disparity attributable not only to men’s greater propensity to select more lethal methods, but also to a higher method-specific lethality even when identical methods are employed (20). Moreover, longitudinal evidence indicates that transitions from non-violent suicide attempts to violent suicide death occur significantly more often among men than among women (3, 21). Our recent cross-sectional analysis of patients treated at two acute care centers in Budapest confirmed previous findings that individuals who attempt violent suicide more closely resemble suicide completers, in terms of demographic and clinical characteristics—including the severity of traumatic injuries—than individuals who use non-violent methods (8, 9). Although violent suicide attempts are associated with substantially higher lethality and case fatality rates, method choice does not reliably reflect suicidal intent, which may be equally high in non-violent attempts (6, 22–24).

Importantly, violent suicide attempts are frequently preceded by prior non-violent behaviors, suggesting a trajectory of escalating lethality and intent over time. A history of suicide attempts is among the strongest predictors of future attempts and completed suicide (25–29). Previous studies suggest the existence of a “cycle of violence,” in which violent suicide attempts and completed suicides are often preceded by earlier violent acts across the lifespan. This pattern reflects elevated lifetime aggression, manifesting in both self-directed and outwardly directed behaviors. Such antecedent behaviors may include self-inflicted injuries such as non-suicidal self-injury, prior suicide attempts, accidental traumatic injuries, as well as aggression toward others (3, 30–34). This pattern supports the notion that exposure to, or engagement in, violent behavior—whether directed toward oneself or others—may reflect an escalating trajectory of risk, culminating in more lethal suicidal behavior (3, 33–35). Recognizing this progression may have important implications for early identification, risk stratification, and targeted prevention strategies. Our previous work (4) supports the hypothesis that severe traumatic injuries and violent suicide attempts are closely interrelated, reflecting a shared vulnerability profile characterized by overlapping risk factors and demographic–clinical patterns typical of trauma-care populations, particularly young to middle-aged men and individuals presenting with alcohol or illicit drug intoxication and significant psychosocial stressors (36–43). Furthermore, severe traumatic injuries—including survival of a prior violent suicide attempt, which itself constitutes a major traumatic event—and their long-term somatic, psychological, and social sequelae may independently contribute to an elevated risk of subsequent suicidal behavior. This risk may be mediated through persistent disability, psychopathological symptoms (e.g., depression, anxiety, post-traumatic stress disorder and alcohol abuse), chronic pain, medication dependence (particularly analgesics and anxiolytics), ongoing psychosocial stressors, and impaired quality of life (44–49). Numerous studies have demonstrated that psychiatric comorbidities—particularly major depressive disorder, personality disorders, and substance use disorders—are significantly associated with repeated suicidal behavior (25, 28, 50–53). Individuals with bipolar disorder, borderline or antisocial personality traits, as well as patients experiencing psychotic episodes are overrepresented among those who engage in recurrent (violent) suicide attempts (54–59). Comorbid substance use further increases the risk of recurrence by exacerbating impulsivity and impairing cognitive control (60–62).

From a neurobiological standpoint, violent suicidal behavior—especially when recurrent—has been linked to serotonergic dysfunction and heightened stress reactivity, underscoring the role of neurochemical and neuroendocrine dysregulation (63, 64). Sociodemographic factors such as age (25–49 years), marital dissolution, unemployment, and lower socioeconomic status have also been identified as significant risk indicators for repeated suicide attempts (25, 65–67). These vulnerabilities often intersect with chronic structural disadvantages—including economic insecurity, housing instability, and lack of social support—frequently culminating in repeated suicidal crises that necessitate trauma care and psychiatric intervention (11, 68).

Collectively, these findings highlight the multifactorial etiology of repeated suicidal behavior, particularly when violent methods are involved. Although evidence suggests that violent and non-violent suicide attempts may represent distinct clinical trajectories, the existing literature on repeated violent suicide attempts remains extremely limited. Most studies examine repetition either in mixed samples of violent and non-violent multiple attempters, without distinguishing between these subgroups, or focus solely on non-violent repeaters. This gap likely reflects the difficulty of accessing such populations and the high lethality associated with violent suicide attempts. Improved delineation of these subgroups may enhance risk stratification and inform more targeted preventive interventions.

In light of the limited data on repeated violent suicide attempts—especially within Hungarian acute trauma settings—the present study addresses this gap through a retrospective analysis of cases treated at a major trauma center in central Budapest. Using electronic health records, we examine demographic, clinical, and method-specific characteristics to identify predictors of repeated violent attempts. Elucidating these patterns is essential for improving clinical risk management and informing evidence-based public health interventions in Hungary and comparable contexts.

Repeated violent suicide attempts appear to represent a clinically distinct, high-risk subgroup characterized by increased somatic and psychiatric complexity and elevated mortality risk. If selective mechanisms are operative—such as a disproportionate burden of specific psychiatric comorbidities—this population may require clinical approaches that differ from those used for individuals with single attempts. Moreover, repeated violent attempts may function as proximal indicators of suicide completion, highlighting the importance of early identification and the implementation of intensive, individualized preventive strategies.

## Methods

### Sample

The sample of violent suicide attempters consisted of patients consecutively admitted for medical treatment to the Dr. Manninger Jenő National Trauma Centre, a level III trauma facility in Budapest, between January 1, 2015, and December 31, 2024. Data were extracted from the institution’s electronic health records covering this specified period. To ensure diagnostic specificity and alignment with the study’s aims, individuals presenting with non-suicidal self-injurious (NSSI) behavior—characterized by deliberate self-harm lacking clear suicidal intent—were excluded from the final analytic sample; these individuals were predominantly not admitted for inpatient care and instead received outpatient trauma care. All included individuals received treatment for confirmed violent suicide attempts. Cases were classified as intentional self-harm based on converging evidence, including suicide notes, eyewitness accounts, collateral information, clinical evaluation, consistency of the injury mechanism, and the exclusion of third-party involvement. Accordingly, these events were not considered accidental traumatic injuries. Comprehensive psychiatric evaluations were conducted using the Structured Clinical Interview for DSM-IV (SCID; 69), administered by board-certified psychiatrists and trained clinical psychologists to establish current and lifetime psychiatric diagnoses. Diagnostic assessments were further corroborated according to the International Classification of Diseases, 10th Revision (ICD-10; 70). Assessment of suicidal behavior, intent, and underlying motivational factors was not limited to the SCID itself but was based on a structured clinical suicide interview conducted by the same trained clinicians. Presence or absence of prior suicide attempts was coded from structured interview and electronic health file records. This interview systematically explored whether the index act constituted a suicide attempt, the individual’s intent to die at the time of the act, previous suicide attempt(s) and the primary motivational drivers (e.g., desire to die, relief from psychological distress, job loss, interpersonal or situational precipitants). While the SCID includes items related to suicidal ideation and behavior within certain diagnostic modules, these were supplemented by targeted, clinician-led inquiries to ensure a comprehensive evaluation of suicide intent and motivation. In addition, motivational factors and method-related variables were systematically categorized based on structured clinical documentation and chart review, using predefined categories to ensure consistency across cases. All assessments followed a standardized clinical framework routinely used in the participating clinical settings and were documented in the medical records. All assessments and diagnostic procedures were carried out at the time of the individual’s admission to the emergency department, once the patient’s somatic condition had stabilized and they were conscious and cognitively accessible for evaluation. In addition, information from next of kin was routinely collected when available.

### Measures

Logistic regression analysis was employed to examine the factors associated with the occurrence of repeated violent suicide attempts. The dependent variable was binary-coded: 0 indicated the absence of a repeated violent attempt, while 1 denoted its presence.

### Statistical analysis

Statistical analyses were conducted using the R statistical software (71). Descriptive analyses were first conducted to characterize the demographic and clinical features of the sample, with continuous variables summarized as means and standard deviations and categorical variables reported as proportions. The descriptive statistics of the data are presented in **Table 1**. These analyses were stratified according to history of suicide attempts. Bivariate associations between established suicide risk factors and history of suicide attempts were assessed using Pearson’s chi-square tests. Multivariate logistic regression models were subsequently constructed to evaluate associations between selected risk factors and repeated violent suicide attempt(s), while adjusting for psycho-socio-demographic covariates, including age, sex, psychiatric diagnosis, method of attempt, and motivational factors. In these models, age (entered as a continuous variable) and sex were controlled for separately to isolate the independent effects of psychiatric variables. Finally, *post hoc* comparisons were conducted using the adjusted Wald test, where appropriate.

**Table 1:** Prevalence of risk factors by repeated violent suicide attempts, disaggregated by gender and in total (N = 327).

Variables	Female			Male			Sum		
	No	Yes	Sum	No	Yes	Sum	No	Yes	Sum
Repeated violent suicide attempt	86	19	105	200	22	222	286	41	327
Organic disorders	100	5	105	221	1	222	321	6	327
Substance abuse	91	14	105	174	48	222	265	62	327
Psychotic disorders	86	19	105	176	46	222	262	65	327
Mood disorders	57	48	105	135	87	222	192	135	327
Anxiety disorders	78	27	105	155	67	222	233	94	327
Behavioral disorder†	104	1	105	222	0	222	326	1	327
Personality disorder	99	6	105	215	7	222	314	13	327
Intellectual disability†	105	0	105	222	0	222	327	0	327
Developmental disorder†	105	0	105	222	0	222	327	0	327
Child and adolescent psychiatric disorder	103	2	105	219	3	222	322	5	327
Crisis (F43.20)	80	25	105	177	45	222	257	70	327
ALCOHOL*	90	15	105	143	79	222	233	94	327
SUBSTANCE*	96	9	105	184	38	222	280	47	327
DRUG*	96	9	105	206	16	222	302	25	327
DEP** (ALC, SUBSTANCE, DRUG) any of them	83	22	105	125	97	222	208	119	327

*Data not derived from a psychiatric diagnosis.

**If any form of dependence on alcohol, drugs, or medication is present.

†A variable excluded from the regression due to lack of occurrence.

## Results

### Sample characteristics

The initial dataset comprised 380 cases of violent suicide attempts treated at a trauma center. Of these, 45 individuals (12%) died by suicide following hospital admission due to the severity of their injuries. Among suicide completers, 76% were male and 24% were female.

Psychiatric history was unavailable for 53 of the 380 cases, primarily due to limited data access. This most commonly resulted from unknown patient identity, missing national health insurance identification numbers, absence of next of kin, or presentation during an acute crisis with underlying, either untreated or subthreshold psychiatric conditions. Because information regarding prior and repeated violent suicide attempts was also unavailable in these cases, key explanatory variables could not be assessed, and these individuals were excluded from subsequent analyses. The final analytic sample therefore consisted of 327 individuals (222 males and 105 females), with a mean age of 46.95 years (SD = 19.5) at the time of admission, of whom 41 were repeat attempters.

### Patterns of suicide attempts and method transition

Among individuals with documented histories, 37% of those with a prior violent suicide attempt subsequently engaged in another violent attempt. Accordingly, among the 55% of violent attempts for which information on prior attempts was available, more than half reflected either repetition of violent methods or escalation from non-violent to violent behavior. Notably, a transition from a prior non-violent attempt to a subsequent violent method was documented in 31% of violent suicide attempters.

### Sex differences in repeated and violent suicide attempts

Among female participants, 18% had a history of repeated (two or more) violent suicide attempts, compared with 10% among male participants. However, in statistical models restricted to individuals with repeated suicide attempts (RSA > 0), sex differences emerged in method choice. A negative regression coefficient for female sex indicated a lower likelihood of violent method use among women and, correspondingly, a higher likelihood among men. The odds ratio for females compared with males was approximately 0.33, with the reciprocal estimate (male vs. female) indicating nearly threefold higher odds of transitioning from a non-violent to a violent method among males.

Consistent with these findings, among individuals with repeated suicide attempts, males were significantly more likely to employ violent methods than females, independent of age. No additional predictors were included in these models due to the limited sample size, which constrained the number of variables that could be reliably entered. Importantly, the effect of sex remained significant after adjustment for psychiatric comorbidity, supporting the robustness of this association.

### Motives, methods and lethality

Among individuals with multiple suicide attempts for whom motive data were available, psychotic symptoms were the most frequently reported precipitating factor, while relationship and intrafamilial conflicts were the most commonly reported motivational factors. Existential crises and chronic and/or severe physical illness were equally prevalent as the third most commonly reported motives.

Among the fatal cases (n = 45), the vast majority (96%) resulted in death at the first suicide attempt (n = 43). Of the individuals who died by suicide, 76% were male. Only two individuals died following multiple suicide attempts, with the third attempt proving fatal in both cases. Stabbing was the most frequently employed suicide method in the overall sample, whereas jumping from a height was associated with the highest lethality.

### Predictors of repeated violent suicide attempts

In the overall sample, a history of a prior violent suicide attempt was associated with a markedly increased likelihood of repeated suicide attempts (OR ≈ 660, 95% CI: approximately 89 to >80,000; p < 0.000001), independent of age and sex, identifying prior violent behavior as the strongest predictor of repetition in the present cohort.

### Psychiatric conditions, comorbidity and repeated violent suicide attempts

Several psychiatric background variables were significantly associated with the likelihood of repeated violent suicide attempts. Substance use disorders (ICD-10: F10–F19) were associated with a significantly increased risk of repetition (OR = 2.86, p = 0.005), as was dependence on sedative or hypnotic medications (MEDICATION; OR = 3.72, p = 0.009), each corresponding to more than a twofold increase in odds. Personality disorders (ICD-10: F60–F69) demonstrated the strongest association, with affected individuals exhibiting more than fourfold higher odds of repeated violent suicide attempts compared with those without such diagnoses (OR = 4.15, p = 0.028). Alcohol abuse alone did not reach statistical significance (p = 0.094), although the observed trend underscored the relevance of substance-related factors. Notably, the co-occurrence of personality disorder and alcohol dependence was associated with an approximately 1.8-fold increase in risk (ALCOHOL coefficient = 0.584, OR ≈ 1.79, 95% CI: 1.00–3.19, p = 0.049), representing a borderline yet statistically defensible effect after controlling for personality disorder. The interaction between personality disorder and illicit drug dependence did not reach statistical significance (DRUG coefficient = 0.34, OR ≈ 1.41, p = 0.38), indicating no detectable joint effect. In contrast, the co-occurrence of personality disorder and medication dependence was strongly associated with increased risk, conferring an approximately fivefold elevation in odds (MEDICATION coefficient = 1.62, OR ≈ 5.05, 95% CI: 2.16–11.97, p < 0.001), reflecting a robust and clinically meaningful relationship.

To account for the rarity of the outcome and the presence of small cell counts, Firth penalized logistic regression was applied using a composite dependence index. In this model, concurrent substance dependence (dep_cluster) was significantly associated with repeated violent suicide attempts (OR = 3.97, 95% CI: 1.87–8.40, p = 0.0004). Polytoxicomania—defined as dependence on at least two substances (alcohol, illicit drugs, or medications)—was associated with nearly fourfold higher odds of repetition, independent of age and sex.

Other psychiatric diagnostic categories, including mood disorders, anxiety disorders, and psychotic spectrum disorders, did not show statistically significant associations with repeated violent suicide attempts. Diagnostic groups with very low prevalence, such as intellectual disabilities and neurodevelopmental disorders, could not be reliably evaluated due to insufficient case numbers.

### Sex-stratified analyses

Sex-stratified models revealed differential risk profiles. Among men, substance use disorders (OR = 2.61, p = 0.024), personality disorders (OR = 6.04, p = 0.0265), and medication dependence (MEDICATION; OR = 3.75, p = 0.0269) were significantly associated with repeated violent suicide attempts. Although psychotic disorders and illicit drug use (DRUG) showed positive associations in direction, neither reached statistical significance (p = 0.1160 and p = 0.0751, respectively).

In contrast, within the female subsample, only substance use demonstrated a notable association with repeated violent suicide attempts (OR = 3.82), although this effect did not reach statistical significance (p = 0.099). **Table 2** shows the risk factors for repeated violent suicide attempts.

**Table 2:** Logistic regression of repeat violent suicide attempt by sex and overall: OR (95% CI) and p-values.

Variables	Base model	P-value	Adjusted model	P-value	Full model	P-value
Men						
Organic disorders	0,00 (0,00–Inf)	0,989	0,00 (0,00–Inf)	0,988	0,00 (0,00–Inf)	0,992
Substance abuse	**2,58 (1,12–5,92)**	**0,026**	**2,61 (1,13–6,02)**	**0,024**	2,12 (0,59–7,63)	0,250
Psychotic disorders	1,90 (0,80–4,50)	0,147	2,07 (0,84–5,10)	0,116	2,64 (0,85–8,13)	0,092
Mood disorders	0,79 (0,35–1,80)	0,578	0,70 (0,28–1,74)	0,436	0,89 (0,30–2,68)	0,838
Anxiety disorders	0,87 (0,36–2,07)	0,744	0,88 (0,37–2,12)	0,775	1,28 (0,42–3,88)	0,664
Personality disorder	**5,45 (1,15–25,74)**	**0,032**	**6,04 (1,23–29,61)**	**0,026**	**7,88 (1,29–48,08)**	**0,025**
Alcohol dependency	1,56 (0,71–3,44)	0,268	1,57 (0,71–3,46)	0,265	0,90 (0,29–2,83)	0,859
Substance	2,07 (0,84–5,10)	0,114	2,46 (0,91–6,60)	0,075	0,92 (0,25–3,37)	0,896
Drug	**3,45 (1,10–10,77)**	**0,033**	**3,75 (1,16–12,10)**	**0,027**	3,30 (0,82–13,38)	0,094
Women						
Organic disorders	2,87 (0,29–28,89)	0,370	5,35 (0,34–84,46)	0,234	7,56 (0,35–162,39)	0,196
Substance abuse	3,86 (0,84–17,69)	0,082	3,82 (0,77–18,82)	0,100	5,56 (0,47–66,28)	0,174
Psychotic disorders	0,00 (0,00–Inf)	0,991	0,00 (0,00–Inf)	0,991	0,00 (0,00–Inf)	0,994
Mood disorders	1,54 (0,39–6,09)	0,538	1,60 (0,40–6,42)	0,504	1,60 (0,28–9,19)	0,600
Anxiety disorders	0,81 (0,16–4,17)	0,802	0,77 (0,15–4,03)	0,761	0,42 (0,04–4,12)	0,458
Personality disorder	2,27 (0,24–21,92)	0,477	2,13 (0,22–21,02)	0,516	3,07 (0,23–40,18)	0,393
Alcohol dependency	3,50 (0,77–15,88)	0,104	3,40 (0,74–15,59)	0,115	0,90 (0,07–11,20)	0,934
Substance	1,37 (0,15–12,43)	0,777	1,21 (0,13–11,80)	0,867	0,61 (0,03–11,54)	0,739
Drug	3,63 (0,63–20,90)	0,148	3,49 (0,56–21,87)	0,181	3,20 (0,17–60,83)	0,440
Total						
Organic disorders	1,54 (0,17–13,50)	0,699	2,02 (0,22–18,73)	0,535	3,23 (0,33–31,50)	0,313
Substance abuse	**2,93 (1,41–6,07)**	**0,004**	**2,82 (1,35–5,87)**	**0,006**	2,53 (0,85–7,51)	0,094
Psychotic disorders	1,29 (0,58–2,88)	0,533	1,27 (0,57–2,85)	0,557	1,48 (0,55–3,95)	0,436
Mood disorders	0,92 (0,46–1,83)	0,809	0,94 (0,47–1,89)	0,866	1,23 (0,52–2,90)	0,641
Anxiety disorders	0,87 (0,41–1,87)	0,725	0,85 (0,40–1,84)	0,685	0,96 (0,37–2,44)	0,924
Personality disorder	**3,66 (1,07–12,53)**	**0,039**	**4,00 (1,15–13,91)**	**0,029**	**4,79 (1,23–18,67)**	**0,024**
Alcohol dependency	1,97 (0,98–3,94)	0,056	1,84 (0,90–3,74)	0,095	1,01 (0,36–2,80)	0,987
Substance	2,05 (0,90–4,66)	0,087	1,94 (0,85–4,44)	0,117	0,75 (0,25–2,29)	0,616
Drug	**3,40 (1,32–8,78)**	**0,011**	**3,50 (1,35–9,10)**	**0,010**	3,01 (0,93–9,81)	**0,067**

Entries show odds ratios (OR) with 95% confidence intervals (CI) and p-values. Base model: univariable. Adjusted model: adjusted for age (continuous); in the total sample additionally adjusted for sex. Full model: all variables entered simultaneously (plus the same adjustment terms). 0.00–Inf values indicate quasi-complete separation; estimates are unstable. Firth penalized logistic regression estimates available upon request.

Bold values means significant values.

### Sensitivity analysis

Given the limited sample size (n = 327) and the relatively low number of repeated-attempt events (n = 41), sensitivity and complementary analyses were conducted using Firth’s bias-reduced logistic regression, implemented via the logistf package in the R programming environment (72). The resulting estimates and statistically significant explanatory variables were consistent with those obtained from standard logistic regression models. A robustness check using discrete age categories (<30, 30–44, 45–59, ≥60), in addition to age as a continuous variable, did not materially alter the results.

## Discussion

### Principal findings

This retrospective clinical study is the first in Hungary to systematically examine patterns and predictors of repeated violent suicide attempts within a high-acuity trauma care setting, a highly specific population that has been only rarely addressed in international research. The findings align with and extend the well-established gender paradox in suicidal behavior, while also highlighting distinct trajectories and risk constellations associated with violent method use and repetition.

Consistent with prior literature (6, 16–19), men were disproportionately represented among individuals employing violent methods and among suicide completers, with 76% of deaths occurring in males. Although 12% of the cohort died by suicide during the study period, the predominance of violent methods among completers mirrors national Hungarian data, where over 85% of suicides involve violent means (12, 15). These findings further support evidence that men are more likely to use highly lethal methods, resulting in severe (neuro)traumatic injuries, higher suicide mortality, and more frequent presentation to acute trauma services. In contrast, our earlier work demonstrated that women more commonly employ non-violent methods and are overrepresented in psychiatric inpatient settings rather than trauma units (8, 9). Together, these findings illustrate an institutional and clinical manifestation of the gender paradox, whereby women more often present to psychiatric care following non-violent attempts, whereas men are more frequently treated in traumatology services after violent attempts.

Importantly, a substantial proportion of individuals initially attempted suicide using non-violent means before later transitioning to violent methods, supporting a trajectory-based model of suicidal behavior in which method severity escalates over time. Male sex was associated not only with violent method use but also with method escalation among individuals with repeated attempts, independent of age. As indicated in the Introduction, longitudinal studies suggest that men are more likely to progress from non-violent suicide attempts to death by violent methods; however, no sex differences in fatal outcomes were observed among individuals exhibiting method transitions in the present sample. This likely reflects limited statistical power and the hospital-based design, which predominantly captures surviving attempters. Accordingly, the observed sex difference in method escalation may represent an earlier stage along the pathway toward more lethal suicidal behavior and a critical window for intervention.

Beyond sex differences, repetition was common among individuals with documented attempt histories, with evidence of both persistence of violent methods and escalation from non-violent to violent behavior. A prior violent suicide attempt emerged as by far the strongest predictor of repetition (OR ≈ 660, p < 0.000001), underscoring its role as a dominant life-course risk marker rather than a transient response to an isolated crisis. In this context, the risk of repetition may be exceptionally high due to the devastating consequences of surviving a prior violent suicide attempt. Several psychiatric conditions were also robustly associated with repeated violent suicide attempts, including polytoxicomania (OR = 3.97), sedative/hypnotic dependence (OR = 3.72), substance use disorders (OR = 2.86), and personality disorders (OR = 4.15), with particularly elevated odds among men. These findings are consistent with extensive prior literature linking psychiatric comorbidity, impulsivity, and substance use to recurrent suicidal behavior (25, 50, 60, 61, 63, 66, 67, 73).

Although alcohol use disorder was highly prevalent in this population, it did not independently predict repeated violent suicide attempts. This likely reflects limited statistical power, diagnostic overlap with other substance-related conditions—particularly polytoxicomania—and heterogeneity in alcohol involvement (e.g., acute intoxication versus chronic disorder). Accordingly, alcohol use disorder appears to function primarily as a contextual or state-dependent facilitating factor, operating through disinhibition and impaired judgment rather than as a stable, independent risk marker.

After controlling for personality disorder, alcohol and medication dependence were associated with an increased likelihood of repetition, whereas drug dependence showed no significant joint effect. Importantly, the strongest association was not linked to any single diagnostic pairing but to the clustering of dependencies. This cluster effect, reflecting polytoxicomania, remained robust (OR ≈ 4) even after adjustment for prior violent suicide attempts, consistent with our earlier findings and highlighting the cumulative impact of multiple substance-related dependencies.

Among psychiatric factors, polytoxicomania exerted a particularly strong and independent effect. From a clinical–theoretical perspective, this association likely reflects a cluster phenomenon rather than an additive one: the concurrent use of multiple substances signals profound impairment in self-regulatory capacity, heightened impulsivity, behavioral disorganization, and compromised inhibitory control during crises. In line with this interpretation, acute psychotic symptoms emerged as the most frequent proximal precipitants of repeated violent suicide attempts—often in the context of substance intoxication or withdrawal—followed by relational conflict, existential crisis, and chronic illness. Notably, repetition was associated with acute psychotic states rather than with primary psychotic disorders as enduring diagnostic entities.

Psychotic symptoms warrant heightened clinical vigilance for suicidal behavior and prevention (74, 75). Acute psychotic phenomena, including hallucinations, delusions, impaired reality testing, and cognitive disorganization, may substantially lower the threshold for violent suicidal behavior by reducing ambivalence, impairing judgment, and amplifying emotional and perceptual distress. Accordingly, psychotic symptoms appear to function less as motivational drivers and more as proximal clinical mediators through which substance-related dysregulation translates into repeated violent suicidal behavior. The frequent co-occurrence of personality disorders and substance misuse likely reflects shared vulnerabilities such as affective instability, impulsivity, and heightened stress sensitivity, which further amplify this risk constellation.

The prognostic significance of prior violent suicide attempts cannot be understood independently of their somatic and social consequences. Survivors frequently sustain severe injuries—such as traumatic brain injury, spinal cord damage, limb amputation, chronic pain, or permanent disability—that may themselves contribute to enduring psychological distress, loss of autonomy, unemployment, social marginalization, and increased reliance on psychoactive substances. Neurocognitive sequelae of traumatic brain injury may further impair executive functioning, impulse control, and emotional regulation, creating a self-reinforcing biopsychosocial vulnerability in the absence of specialized neuropsychiatric follow-up and comprehensive rehabilitation (47, 48, 76, 77).

Finally, a substantial proportion of individuals engaging in violent suicide attempts had prior presentations to traumatology services for accidental injuries or violent acts, frequently occurring under conditions of alcohol intoxication and substance abuse (e.g., intoxicated driving–related accidents, falls while intoxicated, domestic violence, street fights, assaults or homicide) (78, 79). These patterns suggest overlapping vulnerability profiles for severe accidental trauma and violent suicidal behavior, characterized by impulsivity, substance misuse, impaired judgment, and high-risk lifestyles (4). Although causal pathways cannot be established from the present data, traumatic injuries and violent acts may represent early warning signs of escalating suicide risk and critical opportunities for preventive intervention. Future longitudinal studies integrating trauma, psychiatric, and mortality data are needed to clarify these pathways and to inform targeted, stage-specific prevention strategies.

These findings have important clinical and public health implications for suicide prevention. Prior traumatic injuries—even seemingly minor events such as fractures—may serve as early warning signs of underlying emotional dysregulation or acute psychological crises, particularly when occurring in the context of alcohol or substance misuse, and should not be viewed solely as isolated somatic events. Individuals presenting with violent suicide attempts—especially those with a history of prior violent attempts, polytoxicomania, personality pathology, or acute psychotic symptoms—constitute a distinctly high-risk subgroup requiring intensive, multidisciplinary intervention. Trauma and emergency care settings therefore represent critical opportunities for early identification, as such individuals often present before sustained engagement with psychiatric services. Accordingly, risk assessment should explicitly incorporate prior traumatic injuries, suicide attempt histories, method trajectories, and patterns of substance use, rather than relying solely on diagnostic categories. Sex-specific risk profiles further indicate that prevention strategies should prioritize method-related risk and escalation potential over attempt frequency alone.

### Strengths and limitations

This study has several notable strengths. First, it draws on real-world clinical data from a high-acuity trauma care setting, capturing a population of violent suicide attempters that is underrepresented in psychiatric and population-based research. Second, by focusing specifically on repeated violent suicide attempts rather than suicide attempts in general, the study was able to identify distinct behavioral trajectories and risk profiles with direct clinical relevance. Third, the application of Firth penalized logistic regression appropriately addressed rare-event bias and small cell counts, ensuring stable and conservative effect estimates. Finally, the integration of demographic, psychiatric, and method-related variables enabled a trajectory-oriented analysis of suicidal behavior that is highly relevant for emergency, trauma, and consultation-liaison psychiatric practice.

Some limitations should also be also acknowledged. The retrospective, hospital-based design restricts causal inference and limits generalizability to suicides occurring outside clinical settings, potentially underestimating sex differences in fatal outcomes. Missing data on psychiatric history and prior suicide attempts reduced the analytic sample and may reflect untreated psychiatric morbidity among first-time attempters. Sample size constraints—particularly in subgroup and sex-stratified analyses—limited statistical power and the number of predictors that could be reliably included in multivariable models. In addition, the absence of detailed information on injury severity, substance dosage, rescue timing, and prehospital circumstances precluded examination of method-specific lethality and survival mechanisms.

## Conclusions

The present study confirms that a prior suicide attempt represents the strongest predictor of subsequent attempts. Beyond identifying key risk markers, the present findings underscore the critical importance of early and sustained psychiatric and psychological care for survivors of violent suicide attempts, who often sustain severe traumatic injuries and develop persistent somatic conditions (e.g., stomas). Psychiatric evaluation, psychological first aid, and suicide risk assessment should be initiated already during the acute care phase, including intensive care unit (ICU) treatment, rather than being deferred until somatic stabilization alone has been achieved. Persistent suicidal intent, a continued wish to die, and early psychopathological reactions—such as acute stress disorder—may significantly hinder physical recovery and treatment adherence. For example, refusal to eat, limited engagement in physiotherapy, or resistance to mobilization may reflect ongoing suicidal ideation rather than purely medical factors, depressive symptoms or motivational barriers. Previous studies have identified chronic somatic conditions as important risk factors for suicide; however, the literature has primarily focused on illnesses such as cancer, epilepsy, and multiple sclerosis, while traumatic injuries are mentioned less prominently and often remain in the background.

Accordingly, survivors of violent suicide attempts should receive regular psychological support and close psychiatric follow-up throughout both the acute and rehabilitation phases of care. Management of these patients requires a complex, multidisciplinary approach involving trauma surgeons, intensivists, psychiatrists, psychologists, rehabilitation specialists, clinical dietitian and nursing staff. Although recovery is often prolonged and clinically challenging, integrated psychiatric and psychological intervention has the potential to improve cooperation with treatment, support functional recovery, and ultimately reduce the risk of subsequent suicide attempts.

## Data Availability

The raw data supporting the conclusions of this article will be made available by the authors, without undue reservation.
